# Outpatient use of antibiotics in uncomplicated diverticulitis decreases hospital admissions

**DOI:** 10.1002/iid3.70031

**Published:** 2024-09-27

**Authors:** Mark Ayoub, Carol Faris, Julton Tomanguillo Chumbe, Nadeem Anwar, Harleen Chela, Ebubekir Daglilar

**Affiliations:** ^1^ Charleston Area Medical Center – West Virginia University Charleston Division, Internal Medicine Department Charleston West Virginia USA; ^2^ Surgery Department Marshall University School of Medicine Huntington West Virginia USA; ^3^ Department of Gastroenterology and Hepatology West Virginia University School of Medicine – Charleston Area Medical Center Charleston West Virginia USA

**Keywords:** Antibiotics, Complications, Diverticulitis, Hospital Admissions, Infection, Outpatient, Surgery

## Abstract

**Introduction:**

Recently, antibiotics use in uncomplicated acute diverticulitis (AD) has been controversial in Europe. The American Gastroenterological Association (AGA) in their 2015 guidelines recommend their selective use. Our study highlights their role in outpatient management.

**Methods:**

We queried the Diamond Network through TriNetX‐Research Network including 92 healthcare organizations. We included large intestine diverticulitis without perforation, abscess or bleeding. Exclusion criteria included any of sepsis criteria, CRP > 15 mg/L, immunodeficiency or HIV, coronary artery disease, chronic kidney disease, history of Crohn's disease or ulcerative colitis, heart failure, hypertension, diabetes or any of the following in the 3 months before study date; clostridium difficile (C. diff) infection, diverticulitis or antibiotics. Patients with AD were divided into two cohorts; patients on antibiotics, and patients not on antibiotics. Cohorts were compared after propensity‐score matching (PSM).

**Results:**

214,277 patients met inclusion criteria. 58.9% received antibiotics, and 41% did not. After PSM, both cohorts had 84,320. Rate of hospital admission was lower in the antibiotic group (3.3% vs 4.2%, *p* < .001). There was a statistical difference between ICU admission (0.1% vs 0.15%, *p* < .01) and the rate of bowel perforation, peritonitis, abscess formation or bleeding (1.3% vs 1.4%, *p* = .044). There was no difference in mortality (0.1% vs 0.1%, *p* = .11), C. diff (0.1% vs 0.1%, *p* = .9), colectomies (0.2% vs 0.2%, *p* = .33), or Acute Kidney Injury (AKI) (0.1% vs 0.1%, *p* = .28).

**Conclusion:**

Outpatient use of antibiotics in patients with uncomplicated AD is associated with lower rates of hospital admissions and complications without changing mortality rate or surgical intervention.

## Graphical abstract

Less costly hospitalizations in the U.S.A. can be achieved with outpatient antibiotic use in mild acute diverticulitis.

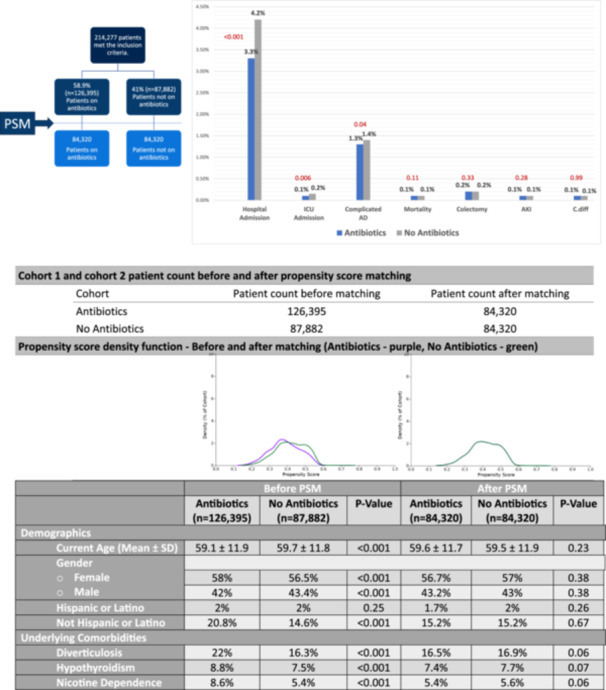

## 1 INTRODUCTION

In up to 25% of patients with diverticular disease, their disease progresses to acute diverticulitis.^1^

Every year, diverticulitis is responsible for more than 2 million outpatient visits and at least 200,000 hospital admissions with an estimated cost of more than 2 billion dollars with an increasing prevalence every year.^2^, ^3^, ^4^ Due to such a high financial burden, attempts to decrease hospital admissions arise. Antibiotics have been the mainstay of treating diverticulitis. Recent studies have been conflicting on their active role in the outcomes of patients with diverticulitis.^5^, ^6^ Some of those studies show no difference in outcomes between the use of antibiotics and conservative management. However, those studies are not of strong evidence or good quality. Also, their statistical significance is questionable. As a result, the American Gastroenterological Association (AGA) updated its guidelines in 2021 and they still recommend the selective use of antibiotics in patients with diverticulitis.^7^ The role of antibiotics is not fully clear and there is no association between certain regimens and outcomes. Historically, antibiotic regimen needs to cover anaerobes and gram‐negative rods. Physicians’ choice of regimen has been relying on such coverage. Since the prevalence of AD is high and the cost of in‐hospital management is increasing, attempts to reduce and prevent hospital admissions are warranted. Therefore, our study aims to address the benefit of antibiotic use in an outpatient setting in the management of acute mild diverticulitis. We also aim to find an association between antibiotic use and different outcomes of the natural course of diverticulitis.

## 2 METHODS

The study was approved by the Institution Board Review Committee at Charleston Area Medical Center. Written informed consent from patients was waived due to the deidentified nature of the TriNetX clinical database. The TriNetX (Cambridge, MA) database is a global federal research network that combines real‐time data with electronic medical records. Our study was conducted using the TriNetX database through the Diamond Network, which comprises of 92 Healthcare Organizations (HCO). Adult patients aged ≥18 years with acute uncomplicated large intestinal diverticulitis who were managed outpatient within the last 20 years, were analyzed. Uncomplicated diverticulitis was defined by the lack of presence of perforation, abscess, or bleeding, as well as absence of systemic infection (temperature >38°C or <36°C, heart rate >90 BPM, respiratory rate >20, leukocytes >12,000/mm³, or <4,000/mm³), immunodeficiency or immunocompromising diseases, history of inflammatory bowel disease, or C‐reactive peptide elevation >15. Patients with mild AD were identified using the codes from the International Classification of Diseases (ICD) −10. The TriNetX database was queried using a full description of study definitions and variables, and their corresponding ICD codes are provided in Table 1.

**Table 1:** Patient Characteristics of Antibiotics Cohort and No Antibiotics Cohort Before and After PSM and Their Corresponding ICD‐10 Codes.

Cohort 1 and cohort 2 patient count before and after propensity score matching						
Cohort	Patient count before matching			Patient count after matching		
Antibiotics	126,395			84,320		
No Antibiotics	87,882			84,320		

Propensity score density function ‐ Before and after matching (Antibiotics ‐ purple, No Antibiotics ‐ green)						
	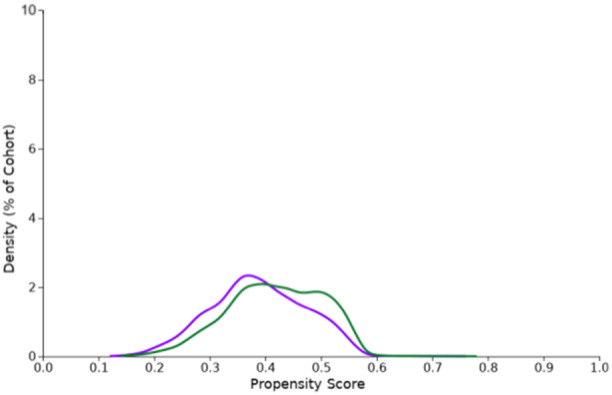			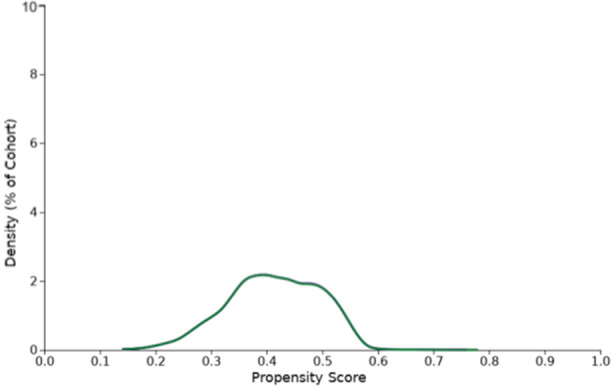		

	Before PSM			After PSM		
	Antibiotics (*n* = 126,395)	No Antibiotics (*n* = 87,882)	*P*‐Value	Antibiotics (*n* = 84,320)	No Antibiotics (n = 84,320)	*P*‐Value
Demographics						
Current Age (Mean ± SD)	59.1 ± 11.9	59.7 ± 11.8	<0.001	59.6 ± 11.7	59.5 ± 11.9	0.23
Gender						
○ Female	58%	56.5%	<0.001	56.7%	57%	0.38
○ Male	42%	43.4%	<0.001	43.2%	43%	0.38
Hispanic or Latino	2%	2%	0.25	1.7%	2%	0.26
Not Hispanic or Latino	20.8%	14.6%	<0.001	15.2%	15.2%	0.67
Underlying Comorbidities						
Diverticulosis	22%	16.3%	<0.001	16.5%	16.9%	0.06
Hypothyroidism	8.8%	7.5%	<0.001	7.4%	7.7%	0.07
Nicotine Dependence	8.6%	5.4%	<0.001	5.4%	5.6%	0.06

Outcome analysis was performed after propensity score matching. Kaplan‐Meier curves and log‐rank tests were used to investigate the differences in all‐cause mortality between groups. Risk Ratios (RR) with 95% confidence intervals (CI) were calculated for each outcome. A *p*‐value of <0.05 was considered statistically significant. All statistical analyses were conducted on the TriNetX platform.

## 3 INCLUSION AND EXCLUSION CRITERIA

Patients with mild uncomplicated acute diverticulitis (AD) who were managed outpatient were identified and divided into two cohorts: patients receiving antibiotics and patients not receiving antibiotics. The antibiotics we used in our analysis were amoxicillin, amoxicillin‐clavulanate, levofloxacin, trimethoprim‐sulfamethoxazole, moxifloxacin, metronidazole, cephalexin, and ciprofloxacin.

We excluded patients with the following comorbidities: hypertension (HTN), coronary artery disease (CAD), heart failure, diabetes mellitus (DM), chronic kidney disease (CKD), history of Crohn's disease or ulcerative colitis, immunodeficiency or HIV. We also excluded patients meeting any of the sepsis criteria including temperature >38°C or <36°C, heart rate >90 BPM, respiratory rate >20, leukocytes >12,000/mm³, or <4,000/mm³, or C‐reactive peptide (CRP) > 15 mg/L. Any patient with history of mild uncomplicated AD within 3 months before diagnosis, or have received the above‐mentioned antibiotics in the same time frame, were also excluded.

The following outcomes were compared within 30 days of diagnosis: hospital admission, intensive care unit (ICU) admission, rate of complicated diverticulitis conversion (presence of perforation, abscess, bleed, or peritonitis), need for colectomy, mortality rate, clostridium difficile (C. diff) infection rate, and acute kidney injury (AKI) rate.

## 4 RESULTS

**Figure 1:**
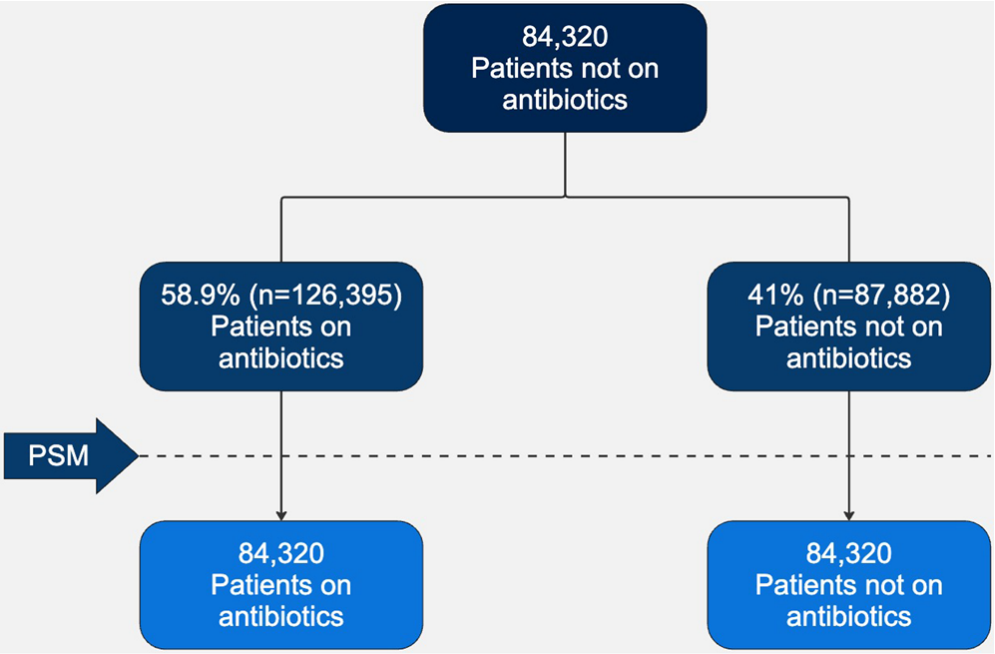
Study Design Flow.

A total of 214,277 patients met our inclusion criteria. Patients with uncomplicated mild AD managed outpatient who received antibiotics (58.9%, *n* = 126,395) and patients with uncomplicated mild AD managed outpatient who did not receive antibiotics (41%, *n* = 87,882). Two well‐matched cohorts of patients receiving antibiotics and those who did not receive antibiotics (*n* = 84,320 vs *n* = 84,320) were compared after propensity‐score matching (PSM) as highlighted in Figure 1.

Before PSM, there was a significant difference between the cohorts’ demographics and comorbidities. PSM was performed to avoid any confounding bias. Analysis of the cohorts’ baseline demographics and comorbidities after PSM did not show any significant difference. The mean age at the index of the antibiotics group was 52.7 with a standard deviation (SD) of 11.7, while in no antibiotics group 52.6 with a SD of 11.9. Slightly more than half the cohorts were comprised of females with 56.7% in the antibiotics group compared to 57% in the no antibiotics group. There were no patients with the diagnosis of heart failure, CAD, CKD, or HTN in either group. Both groups had a similar history of nicotine dependence 5.4% compared to 5.6% and a similar history of diverticulosis 16.5% in the antibiotics group compared to 16.9% in the no antibiotics group. A table highlighting key elements of PSM before and after PSM is highlighted in Table 2.

**Table 2:** Summary of Results.

	Antibiotics (*n* = 84,320)	No Antibiotics (*n* = 84,320)	*P*‐Value
**Hospital Admission**	3.3% (2,781)	4.2% (3,511)	< .001
**ICU Admission**	0.1% (85)	0.15% (125)	.006
**Complicated diverticulitis (perforation, abscess, bleed, or peritonitis)**	1.3% (1,082)	1.4% (1,177)	.044
**Mortality**	0.1% (68)	0.1% (51)	.119
**Clostridium Difficile**	0.1% (97)	0.1% (97)	.99
**Colectomy**	0.2% (182)	0.2% (201)	.33
**AKI**	0.1% (106)	0.1% (122)	.28

**Figure 2:**
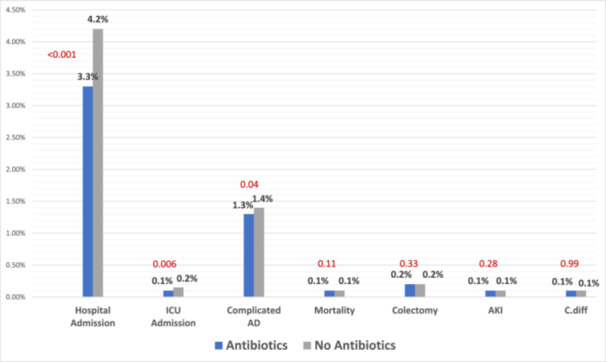
Summary of Results.

After PSM, we compared different outcomes between the two cohorts. Patients who had mild uncomplicated AD who were managed outpatient with antibiotics had a statistically significant lower rate of hospital admissions compared to those who did not receive antibiotics (3.3% vs 4.2%, *p* < .0001). They were also less likely to be admitted to the ICU when compared to those who did not receive antibiotics (0.1% vs 0.15%, *p* = .006). Patients who received antibiotics outpatient also had a lower rate of diverticulitis‐related complications such as perforation, abscess, bleeding, or peritonitis when compared to those who did not receive antibiotics (1.3% vs 1.4%, *p* = .04). However, there was no statistically significant difference in the need of colectomy between the two cohorts (0.2% vs 0.2%, *p* = .33). There was no significant difference in mortality rate in the 30‐days period between the two cohorts (0.1% vs 0.1%, *p* = .11). There was no significant difference in C. diff infection rate (0.1% vs 0.1%, *p* = .99) or the rate of AKI (0.1% vs 0.1%, *p* = .28) between the two cohorts. A full comparison of results as well as its summary are highlighted in Table 3 and Figure 2.

**Table 3:** ICD‐10 and CPT Codes Used in Methodology.

***Codes Used in Methodology (Inclusion, Exclusion, and Outcomes)***		
Outpatient	CPT:99213	
	CPT:99214	
	CPT:99203	
	CPT:99204	
	CPT:99212	
	CPT:99202	
	CPT:99215	
	CPT:99211	
	CPT:99205	
Uncomplicated AD	Diverticulitis of both small and large intestine without perforation or abscess without bleeding	K57.52
	Diverticulitis of large intestine without perforation or abscess without bleeding	K57.32
**Antibiotics**	clavulanate	
	levofloxacin	
	trimethoprim	
	moxifloxacin	
	amoxicillin	
	metronidazole	
	cephalexin	
	sulfamethoxazole	
	ciprofloxacin	
Immunodeficiency	Other immunodeficiencies	D84
CAD	Atherosclerotic heart disease of native coronary artery	I25.1
CKD	Chronic kidney disease (CKD)	N18
Crohn's Disease	Crohn's disease [regional enteritis]	K50
Ulcerative Colitis	Ulcerative colitis	K51
HIV	Human immunodeficiency virus [HIV] disease	B20
Diabetes Mellitus	Diabetes mellitus	E0.8‐E13
	Type 2 diabetes mellitus	E11
Heart Failure	Heart failure	I50
	Heart failure, unspecified	I50.9
HTN	Essential (primary) hypertension	I10
C. diff	Enterocolitis due to Clostridium difficile, recurrent	A04.71
	Enterocolitis due to Clostridium difficile, not specified as recurrent	A04.72
	Enterocolitis due to Clostridium difficile	A04.7
Complicated AD	Diverticulitis of intestine, part unspecified, with perforation and abscess	K57.8
	Diverticulitis of intestine, part unspecified, with perforation and abscess without bleeding	K57.80
	Diverticulitis of intestine, part unspecified, with perforation and abscess with bleeding	K57.81
	Diverticulitis of large intestine with perforation and abscess with bleeding	K57.21
	Diverticulitis of large intestine with perforation and abscess with bleeding	K57.21
	Diverticulitis of large intestine with perforation and abscess without bleeding	K57.20
	Diverticulitis of both small and large intestine without perforation or abscess with bleeding	K57.53
	Diverticulitis of large intestine without perforation or abscess with bleeding	K57.33
	Diverticulitis of large intestine without perforation or abscess without bleeding	K57.32
	Diverticulitis of both small and large intestine with perforation and abscess with bleeding	K57.41
	Diverticulitis of both small and large intestine without perforation or abscess with bleeding	K57.53
	Diverticulitis of large intestine with perforation and abscess	K57.2
	Diverticulitis of both small and large intestine with perforation and abscess	K57.4
	Diverticulitis of both small and large intestine with perforation and abscess without bleeding	K57.40
	Peritonitis	K65
	Peritonitis, unspecified	K65.9
	Peritoneal abscess	K65.1
Hospital Admission	Hospital Inpatient Services	CPT 1013659
ICU Admission	Critical Care Services	CPT 1013729
Colectomy	Colectomy, partial	CPT 1007455
	Colectomy, partial; with anastomosis	CPT 44140
	Colectomy, partial; with end colostomy and closure of distal segment (Hartmann type procedure)	CPT 44143
	Colectomy, partial; with coloproctostomy (low pelvic anastomosis)	CPT 44145
	Colectomy, total, abdominal, without proctectomy	CPT 1007463
	Colectomy, total, abdominal, without proctectomy; with ileostomy or ileoproctostomy	CPT 44150
	Colectomy, partial; with skin level cecostomy or colostomy	CPT 44141
	Colectomy, partial; with resection, with colostomy or ileostomy and creation of mucofistula	CPT 44144
	Colectomy, partial; with coloproctostomy (low pelvic anastomosis), with colostomy	CPT 44146
	Colectomy, total, abdominal, with proctectomy	CPT 1007468
	Colectomy, total, abdominal, with proctectomy; with ileostomy	CPT 44155
	Colectomy, partial; abdominal and transanal approach	CPT 44147
AKI	Acute kidney failure	N17
	Acute kidney failure, unspecified	N17.9
	Acute kidney failure and chronic kidney disease	N17‐N19
***Propensity Score Matching Components***		
Nicotine Dependence	Z87.891	
Diverticulosis	K57.30	
Hypothyroidism	E03.9	

AD: Acute Diverticulitis; CAD: Coronary Artery Disease; CKD: Chronic Kidney Disease; HIV: Human Immunodeficiency Virus; HTN: Hypertension; C. diff: Clostridium Difficile; ICU: Intensive Care Unit; AKI: Acute Kidney Injury.

## 5 BACKGROUND AND DISCUSSION

Acute diverticulitis (AD) is clinically defined as inflammation of a diverticulum or diverticula, typically within the colon. This occurs in up to 25% of patients with history of diverticulosis.^8^ Complications such as abscess formation, perforation, fistula formation, or colonic obstruction, end up happening in up to 15% of those patients. Recurrence of AD is also common in up to 30% of those patients. AD typically presents with constant abdominal pain usually severe and mainly localized to the left lower quadrant. Other signs associated with pain include; fever, change in bowel habits, rectal bleed, or presence of mucous in stool. The Nationwide Inpatient Sample (NIS) revealed that there was 26% increase in diverticulitis‐associated hospitalization with a 38% increase in elective operative management.^8^

Multiple risk factors and comorbidities were associated with a higher risk of AD.^9^, ^10^, ^11^ The risk of AD was found to be 50% attributable to genetic factors with an increasing risk with advancing age.^7^, ^10^ Smoking and obesity both have a significant association with an increased risk of AD and its complications.^12^, ^13^ Hypothyroidism and obesity were associated with a 2.4 times higher risk of AD in a study of 3,175 patients.^14^ Hypertension was associated with a 1.8 times higher risk of AD in a Swedish study of 7,500 patients.^12^ Additionally, hypertension and diabetes mellitus were found in 31% and 22% respectively in a Japanese population with diverticular disease.^15^ A literature review of 25 qualifying studies of AD in immunocompromised patients found an incidence of AD up to 15% in those patients.^16^ Another study showed a significantly increased mortality rate in patients with AD who have HIV infection with an odds ratio of 3.9.^17^ Although there was no direct association between IBD and risk of AD, some gut microbiomata studies showed a decrease in Enterobacteriaceae and Clostridiales families suggesting that such a change in the fecal microbiome driven by low‐grade inflammation is associated with AD.^18^, ^19^, ^20^, ^21^ In two Japanese studies, a higher odds ratio of AD complications was found in patients with coronary artery disease (OR of 1.9‐2.4) and chronic kidney disease (OR of 6.4).^22^, ^23^ This was further confirmed in a British study that found an odds ratio of 18.7 of chronic kidney disease in deceased patients from complicated AD.^24^ A Swedish study of 37,000 patients found a 24% increased risk of hospitalization from AD in females who smoke.^25^ Another Swedish study of 7,500 patients found a 60% increased risk of hospitalization from AD in males who smoke.^12^ The previously mentioned risk factors and comorbidities are used in our inclusion/exclusion criteria as well as propensity‐score matching components to ensure comparable cohorts.

The American Society of Colon and Rectal Surgeons guidelines for management of acute diverticulitis recommend an initial lab evaluation using CRP.^26^ The basis of their recommendation was a retrospective study of 350 patients where they were able to significantly discriminate between acute uncomplicated diverticulitis from complicated diverticulitis.^27^ They also recommend the use of CT scan due to its ability to distinguish complicated from uncomplicated diverticulitis.^26^ As for medical management, they gave strong recommendations about treating without antibiotics in select patients and may be possible outpatient, however, they stated that antibiotics remain the mainstay of treatment even outpatient.^26^ The reported success rate of outpatient management of acute uncomplicated diverticulitis is about 94%–97% using the standard of care including bowel rest, increase fluid intake, and oral antibiotic therapy.^8^

The most recent American Gastroenterological Association (AGA) guidelines recommend the use of antibiotics selectively in patients with AD.^7^, ^28^ The antibiotics were recommended to be used selectively in patients who are immunocompetent with mild uncomplicated AD. They also advised to use antibiotics in patients with uncomplicated disease who have comorbidities, have elevated CRP, have elevated white blood cell count, or have a fluid collection.^7^ Antibiotics should be broad‐spectrum with activity against anaerobes and Gram‐negative rods. Amoxicillin plus clavulanic acid, sulfamethoxazole‐trimethoprim (SMX‐TMP) with metronidazole, or a quinolone with metronidazole meet those criteria. Generally, symptomatic improvement should be apparent within 2–3 days. Antibiotics should be continued for 7–10 days.

Recent studies have been controversial about the role of antibiotics in managing AD. A randomized controlled trial (AVOD trial) in Sweden in 2012 in 623 patients concluded that antibiotic treatment for acute uncomplicated AD neither accelerates nor prevents complications or recurrence.^5^ There have not been many studies involving the use of antibiotics in uncomplicated AD. A systemic review included 5 studies that addressed the use of antibiotics; the first three compared no antibiotics vs antibiotics, the fourth study compared single vs double agent, and the fifth study compared short vs long duration of IV antibiotic therapy. The quality and certainty of the evidence is low for all of those studies.

The first study did not show a difference in the rate of complications in 30 days; however, it showed that the rate of emergent surgeries and the rate of adverse effects were lower in the antibiotics group.^5^, ^6^ The second study did not show a difference in the rate of recurrence or complications or emergency surgery beyond 30 days.^29^, ^30^ The Third study showed that the mortality rate was lower in the antibiotics group.^31^ Fourth study showed that the use of a single agent was associated with a higher cure rate and less adverse effects.^32^ The Fifth study showed no difference between the two groups in adverse effects; however, the short‐duration group was associated with a higher rate of treatment failure.^33^ Conclusion of the above systemic review proves that the evidence on antibiotic treatment for uncomplicated AD is uncertain for complications, urgent surgery, recurrence, and long‐term complications.^34^

The Infectious Disease Society of America (IDSA) has certain recommendations for antibiotic management of extra‐biliary intraabdominal infections. Their single‐agent regimen for mild‐moderate infection in adults: cefoxitin, ertapenem, moxifloxacin, and ticarcillin‐clavulanic acid. In more severe infections, meropenem and piperacillin‐tazobactam can be considered. Their combination agent regimen for mild‐moderate infection in adults include metronidazole used in combination with one of those agents: cefazolin, cefuroxime, ceftriaxone, cefotaxime, ciprofloxacin, or levofloxacin. In a more severe infection, the second agent can be cefepime or ceftazidime. The recommended duration of antimicrobial therapy should be limited to 4–7 days unless source control is not obtained. If that is the case, longer periods can be considered.^35^ The agents for mild‐moderate infections are similar to the ones recommended by the AGA, which are the ones we used in our study.

The outcomes of AD vary depending on the treatment approach; surgical vs medical. Recurrence following an episode of AD varies from 13.3% to 42% depending on diagnostic criteria and follow‐up period.^36^ The largest retrospective series reported a recurrence rate of 13.3% in a median follow‐up of 8.9 years in medically treated patients (8). Risk factors associated with recurrence have not been clearly outlined. Mortality associated with AD also was not clear. The majority of studies have been reporting a reduction in mortality over time. No clear association was made between mortality and treatment modality. The risk and the rate of perforation is not clear when attempting to make an association with each treatment modality. Routine elective colectomy after 2 episodes was considered standard of care but this has changed with time. The DIRECT trial is a randomized comparison of elective resection vs medical management. Results are still pending after the trial was disrupted.^37^ After initiating antibiotics and the initial resuscitation, there has not been a clear association between the chosen treatment and outcomes. There have been studies attempting to make such an association, however, metanalysis of those studies was unable to make such association.^38^

A survey was mailed to fellows of The American Society of Colon and Rectal Surgeons to document the preferred medical treatment for uncomplicated AD. Half of the correspondents used a single IV antibiotic with a second‐generation cephalosporin and ampicillin/sulbactam being the most commonly used ones. The other half reported using a combination therapy with ciprofloxacin/metronidazole and TMP‐SMX/metronidazole being the most commonly used in 28% and 6% respectively. The majority of correspondents prescribed an oral antibiotic on discharge in a regimen of 7‐10 days with ciprofloxacin, amoxicillin/clavulanate, and metronidazole being the most common.^39^ These agents are consistent with the recommendation of IDSA. However, the decision to start antibiotics in patients with mild uncomplicated AD remains selective and made on an individual basis.

Numerous studies show an increasing trend toward hospitalization from AD. One study evaluating patients with diverticulitis in US emergency rooms showed that 60% of patients with diverticulitis were admitted.^40^, ^41^ Another showed a 7.5% growth in the rate of hospitalization for patients with uncomplicated AD in Italy from 2005 to 2015.^42^ Our study shows 0.9% reduction of admission rate in patients with mild uncomplicated AD when treated with antibiotics outpatient. ICU admission is typically reserved for unstable patients with complicated AD, our study also showed a 0.05% reduction of ICU admission with the use of antibiotics. The severity of diverticulitis is usually categorized using the modified Hinchey Classification as in Table 4. Complicated diverticulitis includes stage Ib, II, III, and IV. Only 1‐2% of patients with AD present as stage III or IV and can have up to 20% postoperative mortality rate.^24^, ^43^ The use of antibiotics in our study shows a reduction of complicated‐conversion (perforation, abscess, bleed, or peritonitis) down to 1.3%, however, the mortality rate remained unchanged at 0.1%. Complicated diverticulitis typically requires surgical intervention such as percutaneous drainage or colectomy. Urgent colectomy is usually reserved for patients who fail medical management and/or percutaneous drainage.^44^ One study suggests that medical treatment is usually successful in 97% of patients with uncomplicated AD,^45^ further proving that urgent colectomy might not be indicated.^44^ This was further confirmed by our study that showed patients with mild uncomplicated AD rarely require colectomy in 30 days and the rate of colectomy is unchanged at 0.2% even with the use of antibiotics. Many studies associated C. diff infection with the use of antibiotics.^46^, ^47^, ^48^ They were also linked with the development of AKI, with one study showing the incidence of up to 18% of patients admitted to the hospital who receive IV antibiotics.^49^ However, the rate of both C. diff and AKI in 1 month was not increased with the use of antibiotics staying at 0.1% and 0.1%, respectively. This highlights the safety of outpatient use of antibiotics in the setting of mild uncomplicated AD.^50^

**Table 4:** Modified Hinchey Classification.

Stage	Modified Hinchey classification	CT findings
**0**	Mild clinical diverticulitis	Diverticulum ± colonic wall thickening
**Ia**	Confined pericolonic inflammation/phlegmon	Colonic wall thickening with pericolonic soft tissue changes
**Ib**	Pericolonic/mesocolic abscess	Ia changes + pericolonic/mesocolic abscess
**II**	Pelvic, distant intraabdominal or retroperitoneal abscess	Ia changes + distant abscess (generally deep in the pelvis or in interloop regions)
**III**	Generalized purulent peritonitis	Free gas associated with localized or generalized ascites and possible peritoneal wall thickening
**IV**	Generalized fecal peritonitis	Same findings as III

The lack of statistically significant difference in the rate of mortality or surgery between the two groups can be explained by the mild nature of the disease and the low‐likelihood of severe progression. The significant reduction in hospital admission in patients receiving antibiotics in outpatient settings should provide a significant financial burden reduction without significant effect on mortality.

There are several limitations to our study. First, we used ICD codes to identify patients, therefore we were unable to obtain any imaging such as CT findings that would address the presence of abscess. Second, we only used the antibiotics highlighted in the guidelines, however, other agents could have been used that were not captured. Also, due to the retrospective nature of the study, we were unable to identify the duration of therapy or compliance with the regimen nor use specific hospital admission diagnosis. Using all‐cause hospital admissions can mask other acute problems leading to hospital admissions that may affect our outcomes, however, we attempted to mitigate any possible bias by performing PSM to account for known and unknown covariates ensuring randomization of a very similar patient population and by using a short period of follow‐up from index date to ensure that admission is related to their initial presentation i.e. mild acute diverticulitis. Additionally, we were unable to account for symptomatology due to the ICD‐10 nature of our database which can play a part in the definition of mild uncomplicated diverticulitis, however, we attempted to mitigate this by using very specific inclusion and exclusion criteria similar to the AVOD trial when defining mild uncomplicated diverticulitis as well as only including outpatient encounters, which is an indirect measure of severity of the disease. Another one of our study limitations is the inability to further classify immunodeficiencies which can confound outcomes, however, we attempted to mitigate this by using a generalized ICD‐10 code for immunodeficiency that can capture other diagnoses underneath. We also attempted to mitigate immunodeficiency by using other systemic diseases that can play a part such as HIV, IBD, DM, CKD, and HF. Lastly, we attempted to study the population that received antibiotics and still progressed to have complications; however, this required a complete restructure of the study including inclusion, exclusion, and PSM components which compromises our data and would make association difficult. However, further sub‐analysis is needed to analyze this subset of patients.

One of the strengths of the study is using PSM and using very specific exclusion criteria which allowed us to have very similar and homogenous cohorts and exclude sicker patients, respectively. Another major strength of our study is the large patient population included in our study which makes the power of the findings very strong and increases the ability to generalize these findings on antibiotics use in patients with mild uncomplicated AD.

## 6 CONCLUSION

The outpatient use of antibiotics in patients with mild uncomplicated AD is associated with lower rates of hospital admissions. It also lowers the rate of bowel perforation, peritonitis, abscess formation, or bleeding in those patients, however, does not affect the need for colectomy or mortality. The use of antibiotics was not associated with an increased rate of C. diff or AKI in those patients. Hence, the use of antibiotics in these cases was noted to be beneficial overall.

## AUTHOR CONTRIBUTIONS

**Mark Ayoub**: Investigation; Writing—original draft; Methodology; Visualization; Writing—review and editing; Software; Formal analysis; Data curation. **Carol Faris**: Writing—original draft; Writing—review and editing; Visualization; Project administration; Resources. **Julton Tomanguillo Chumbe**: Investigation; Validation; Methodology; Writing—review and editing; Software; Data curation; Resources. **Nadeem Anwar**: Conceptualization; Investigation; Validation; Writing—review and editing; Project administration; Supervision. **Harleen Chela**: Conceptualization; Investigation; Writing—review and editing; Validation; Supervision; Resources. **Ebubekir Daglilar**: Conceptualization; Investigation; Writing—review and editing; Methodology; Validation; Formal analysis; Project administration; Supervision.

## CONFLICT OF INTEREST STATEMENT

All authors declare that they have no conflicts of interest.

## TRANSPARENCY STATEMENT

The legal and ethical restrictions under which the data were provided do not allow for the data to be made publicly available. The data we used for this paper was acquired from TriNetX (https://www.trinetx.com/). Release and/or sharing of this data are not covered under our data use agreement with TriNetX. To gain access to the data, a request can be made to TriNetX (moc. xtenirt@nioj), but costs may be incurred, and a data sharing agreement would be necessary.

## INSTITUTIONAL REVIEW BOARD STATEMENT

The study was conducted in accordance with the Declaration of Helsinki, and approved by the Institutional Review Board at Charleston Area Medical Center in Charleston, West Virginia. IRB NUMBER: 22‐896 IRB; APPROVAL DATE: 11/22/2022.

## INFORMED CONSENT STATEMENT

Patient consent was waived due to the deidentified nature of the database.

## Supporting information

Supporting information.

## Data Availability

Data Used: We used TriNetX research database.
